# Electrocatalytic and Photocatalytic N_2_ Fixation Using Carbon Catalysts

**DOI:** 10.3390/nano15010065

**Published:** 2025-01-02

**Authors:** Changchun Xu, Hongli Su, Shuaifei Zhao, Azadeh Nilghaz, Kunning Tang, Luxiang Ma, Zhuo Zou

**Affiliations:** 1 School of Electrical and Energy Engineering, Yangzhou University, Yangzhou 225100, China; 2 Resource & Recycling, Department of Engineering Structures, Faculty of Civil Engineering and Geosciences, Delft University of Technology, 2628CN Delft, The Netherlands; h.su-3@tudelft.nl; 3 Institute of Frontier Materials, Deakin University, Waurn Ponds Campus, Geelong, VIC 3220, Australia; s.zhao@deakin.edu.au (S.Z.); a.nilghaz@monash.edu.au (A.N.); 4 School of Minerals and Energy Resources Engineering, The University of New South Wales, Sydney, NSW 2052, Australia; kunning.tang@unsw.edu.au; 5 College of Materials and Chemistry & Chemical Engineering, Chengdu University of Technology, Chengdu 610059, China; maluxiang@cdut.edu.cn; 6 Institute of Materials Science & Devices, School of Materials Science and Engineering, Suzhou University of Science and Technology, Suzhou 215009, China

**Keywords:** N_2_ fixation, carbon catalysts, electrocatalysis, photocatalysis, hydrogen evolution reaction, defective carbon materials

## Abstract

Carbon catalysts have shown promise as an alternative to the currently available energy-intensive approaches for nitrogen fixation (NF) to urea, NH_3_, or related nitrogenous compounds. The primary challenges for NF are the natural inertia of nitrogenous molecules and the competitive hydrogen evolution reaction (HER). Recently, carbon-based materials have made significant progress due to their tunable electronic structure and ease of defect formation. These properties significantly enhance electrocatalytic and photocatalytic nitrogen reduction reaction (NRR) activity. While transition metal-based catalysts have solved the kinetic constraints to activate nitrogen bonds via the donation-back-π approach, there is a problem: the d-orbital electrons of these transition metal atoms tend to generate H-metal bonds, inadvertently amplifying unwanted HER. Because of this, a timely review of defective carbon-based electrocatalysts for NF is imperative. Such a review will succinctly capture recent developments in both experimental and theoretical fields. It will delve into multiple defective engineering approaches to advance the development of ideal carbon-based electrocatalysts and photocatalysts. Furthermore, this review will carefully explore the natural correlation between the structure of these defective carbon-based electrocatalysts and photocatalysts and their NF activity. Finally, novel carbon-based catalysts are introduced to obtain more efficient performance of NF, paving the way for a sustainable future.

## 1. Introduction

**Figure 1:**
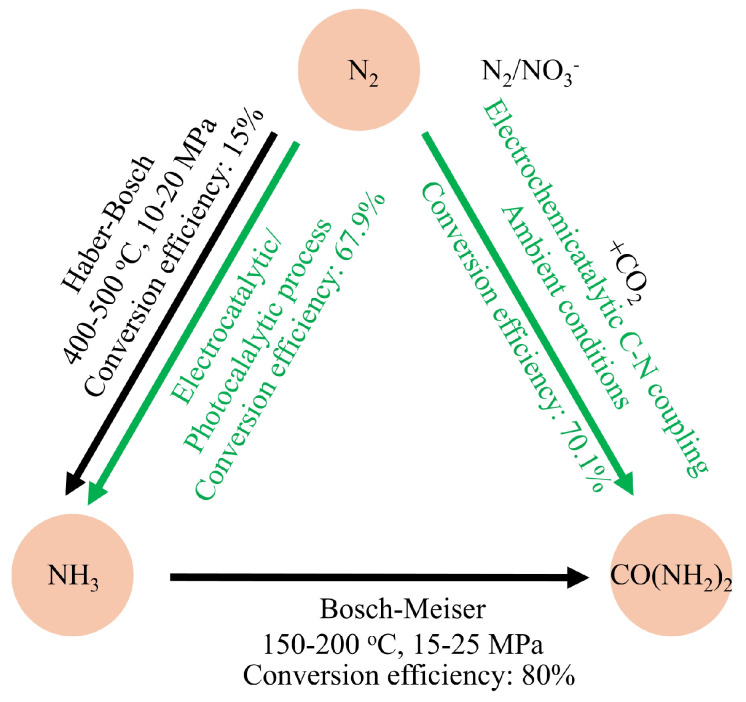
Schematic illustration of the ammonia and urea synthesis scheme in the conventional energy-intensive pathway and the alternative C-N coupling pathway under ambient conditions.

The production of urea and ammonia as precursors of nitrogen fertilizers through nitrogen fixation is of great scientific significance. Currently, the conventional industrial methods for synthesizing urea, ammonia, and other nitrogenous sources are complex and require a lot of energy input (Figure 1) [1,2]. Therefore, it is imperative to study environmentally friendly, sustainable, and efficient urea and ammonia synthesis methods to replace traditional technologies. Ammonia has been employed in agriculture as a nitrogen fertilizer, in emerging green energy, as a vital raw compound for pharmaceuticals, and in hydrogen storage [3,4,5]. In 2022, global ammonia consumption was ~170 M tons according to ChemAnalyst, with around 65% of it consumed in the fertilizer field. Ammonia is mainly converted by nitrogen via Haber–Bosch (H-B) technology, benefiting the majority of the world’s population [6]. In the H-B process, the reaction by converting a mixture of N_2_ and H_2_ to NH_3_ is induced by iron catalysts at high pressure and temperature to overcome the high kinetic barrier associated with the cleavage of N≡N [7,8,9,10,11,12,13]. Nevertheless, only ~15% conversion is obtained in a single process of nitrogen-to-ammonia cycle, even at high pressure (>40 MPa). The unreacted H_2_ and N_2_ are recycled to reach a final overall yield of 97%, occurring at the high pressure and temperature conditions provided by a high energy input [14]. The energy consumed to produce ammonia is ~485 kJ·mol^−1^ in the whole process [15]. Around 452 Mt of CO_2_ is generated in the NH_3_ synthesis process based on the IEA report [16]. In addition, the industrial urea synthesis mainly adopts the Bosch–Meiser process with high temperature and high pressure (150–200 °C, 15–25 MPa). This conventional, energy-intensive route to urea and ammonia synthesis has a significant impact on global energy and environmental challenges. Thus, there is an urgent need to fix nitrogen in an energy-efficient and environmentally friendly manner [17,18].

Davy et al. [19] reported an electrocatalytic nitrogen fixation (NF) method in the 19th century. Since then, the reduction in energy consumption in the NF approach has attracted tremendous research attention [20,21], and significant progress has been made regarding nitrogen fixation via electrocatalytic and photocatalytic approaches. For example, Pickett et al. (1985) first synthesized ammonia by the dinitrogen complex with electricity at room temperature and pressure [22]. Oshikiri et al. (2016) prepared ammonia from atmospheric water and nitrogen under sunlight irradiation to achieve a sustainable energy and low-carbon society [23]. Zhou et al. made ammonia with a high conversion efficiency of 60% for nitrogen by the ionic liquid, which has a high nitrogen solubility [24]. Zheng et al. reported a potentially convenient and green method for NF with a satisfactory conversion efficiency and high yield of ammonia under mild operating conditions [25].

Metal-based catalysts can effectively reduce operating and capital costs by reducing the experimental pressure and temperature [26]. Novel catalysts (e.g., Ru) have demonstrated a low energy barrier due to reactant chemisorption on active centers and the following activation approach caused by electron transfer [27]. For example, Ken et al. (1972) reported that a Ru-based catalyst whose catalytic efficiency was 10 times more than traditional Fe-based catalysts [28]. The possible reason is that the kinetic energy barrier of N≡N in the π-feedback path is reduced, and the empty d-orbital of the Ru-based catalyst receives the lone pair of electrons from nitrogen [29,30]. The occupied d-orbital of the Ru-based catalyst donates electrons to the anti-bonding orbital of nitrogen. It derogates the N≡N bond, resulting in a “donate–accept” reaction path. Although Ru-based catalysts have a relatively good catalytic performance in converting H_2_ and N_2_ to ammonia, their insufficient stability and high cost still hinder their practical application for industrialization [14]. Furthermore, electrons in the d-orbitals of Ru-based catalysts can form H-Ru bonds, leading to undesirable side reactions (hydrogen evolution reactions (HER)) [31]. Research on transition metal-based catalysts has exhibited low faradaic efficiencies (<10%) and generation rates of ammonia (<10^−8^ mol·cm^−2^·s^−1^) [32], while the required faradaic efficiencies for industrialization are over 10% and generation rates of ammonia are around 10^−6^ mol·cm^−2^·s^−1^) [33]. Thus, it is necessary to probe novel catalysts that enable huge changes in electronic structure to achieve the required catalytic performance [34].

**Figure 2:**
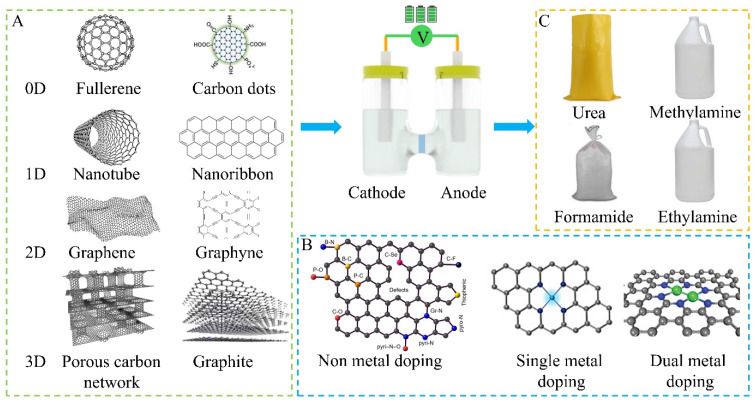
Schematic illustration of carbon catalysts for nitrogen fixation: (**A**) Carbon catalysts ranging from 0 D to 3 D. (**B**) Non-metal and metal doping carbon catalysts. (**C**) C/N products.

Carbon-based catalysts are promising alternatives for metal-based catalysts due to their high specific surface area, tunable defects and porosity, excellent mechanical properties, and optimal electrical conductivity [35]. Various carbon-based catalysts, including graphene, carbon nanotubes, heteroatom-doped carbon dots, single-atom metal-doped carbon, and dual-metal-doped carbon show low-active centers for chemical adsorption of reaction intermediates and/or reactants (Figure 2). Energy sites are clustered near the periphery of the carbon layer [36]. These sites are rich in unpaired electrons can be saturated with heteroatoms or hydrogen and are active centers for reactant dissociation or activation [37]. When graphite sheets have non-hexagonal defects (e.g., octagonal, heptagonal, or pentagonal shapes), the additional charges associated with these defects enhance the conversion of adsorbed molecules, thereby amplifying undesirable reactivity in the substrate plane [38]. Compared to the basal plane in graphite structures, edge areas, and defective units are reduced in nanocarbon with a well-defined crystal structure [39]. Thus, these materials often lack significant heterogeneous catalytic activity and require engineered defects to make them active [40].

In this paper, we present a summary of current advances in the study of NF and the state-of-the-art carbon-based catalysts for NF under ambient conditions. The fabrication and application of defective, heteroatom-doped, and (single-atom and dual-atom) metal-doped carbon-based catalysts in NF are presented. Subsequently, the electrocatalytic and photocatalytic NF by carbon-based catalysts are discussed. Finally, the challenges and outlook in this exciting field are also presented.

## 2. Mechanisms of Nitrogen Fixation

### 2.1. Mechanism of Ammonia Generation

**Figure 3:**
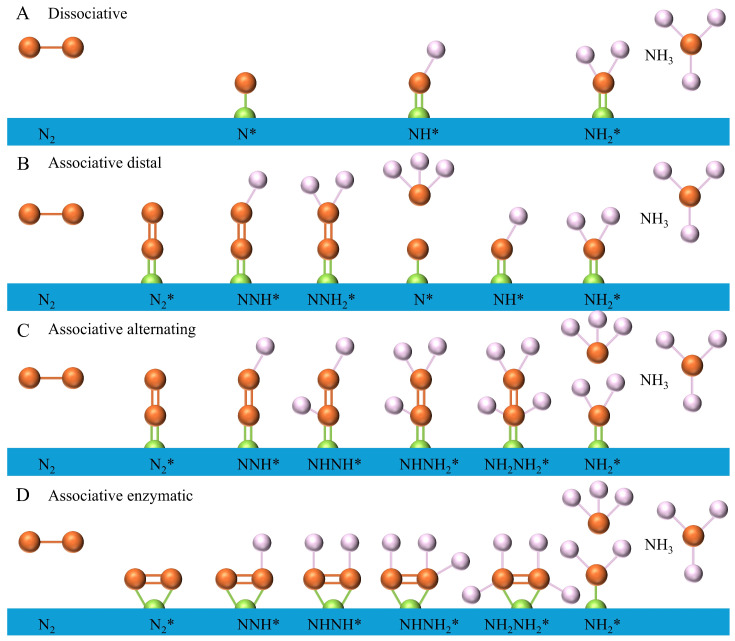
Possible mechanism of ammonia generation by nitrogen. Reprinted with permission from [42], copyright 2023, Wiley-VCH.

Recently, the electrocatalytic synthesis of ammonia has two main mechanisms: dissociative and associative. Figure 3A shows the dissociative approach as *N≡N → N* → NH* → NH_2_* → NH_3_ [41]. The triple bond of nitrogen is broken before hydrogenation. This approach requires much energy to break down the covalent bond of nitrogen. Also, it is limited by the Brønsted–Evans–Polanyi relation. As a comparison, the hydrogenation occurs earlier, which converts nitrogen molecules into NNH*. According to the order of H addition, the hydrogenation process can be divided into distal hydrogenation and alternating hydrogenation [42]. In the distal hydrogenation, the distal nitrogen atom (far away from the end-on adsorption site) is preferentially hydrogenated until the first ammonia molecule is released (Figure 3B). Then, the other N atom repeats this hydrogenation approach to form another ammonia molecule [43]. In the alternating hydrogenation process, in addition to the proton-coupled electron transfer, the two nitrogen atoms in the nitrogen molecule are hydrogenated to form ammonia molecules and are released sequentially (Figure 3C). Also, the enzymatic approach shows a similar hydrogenation approach to the alternating approach (Figure 3D). However, both N atoms are bound to the catalyst surface in a lateral coordination mode. The detailed intermediates that occur in these approaches are shown in Table 1.

### 2.2. Mechanism of C-N Coupling to Form Nitrogenous Compounds

**Figure 4:**
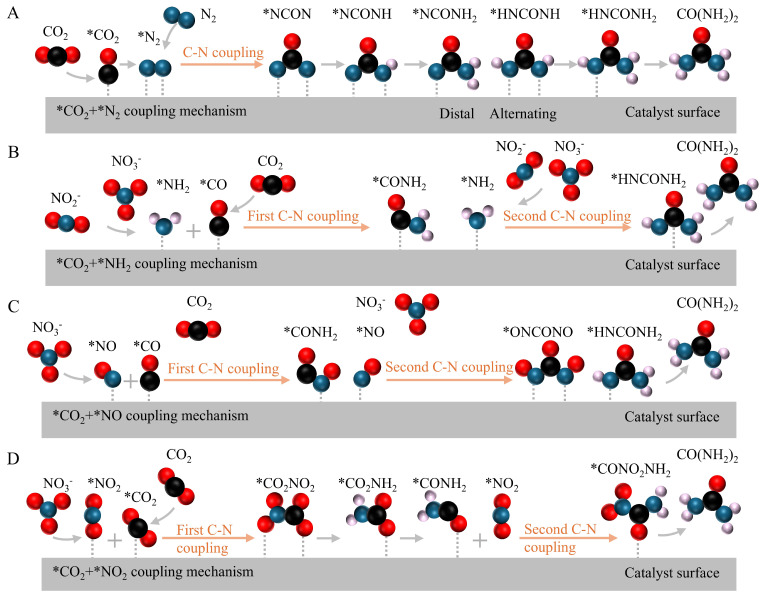
Various coupling mechanisms of urea generation by CO_2_ with various nitrogen sources: (**A**) *CO + *N_2_. (**B**) *CO + *NH_2_. (**C**) *CO + *NO. (**D**) *CO_2_ + *NO_2_. Reprinted with permission from [44], copyright 2024, Wiley-VCH.

The nitrogenous compounds (e.g., methylamine, ethylamine, formamide, and urea) can be generated by C-N coupling from nitrogen and carbon dioxide. In the C-N coupling approaches, nitrogen and carbon dioxide species are first converted into various intermediates, (e.g., *NH_2_, *NH, *H_2_NOH, *NO, *NO_2_, *N_2_, *COOH, *CO, and *CO_2_) (Figure 4). The C-N coupling principles are vital to designing and optimizing catalysts to catalytically synthesize various nitrogenous compounds.

Chen et al. (2020) first reported the C-N coupling for synthesizing urea by simultaneously reducing the N_2_ and CO_2_ [45]. PdCu nanoparticles as the catalysts are deposited on TiO_2_ nanosheets for the electrochemical synthesis of urea. N_2_ and CO_2_ are directly coupled with water molecules for urea formation under ambient conditions. The C-N bonds in urea are formed by the thermodynamically spontaneous reaction between CO and *N=N*. Density functional theoretical calculations and the isotope-labeled operando synchrotron-radiation Fourier transform infrared spectroscopy are used to reveal the reaction approaches, intermediates, and quantification of products. Firstly, nitrogen molecules are absorbed on the surface of catalysts and activated on these catalytic active sites to form *N_2_. CO_2_ molecules are converted to *CO on the adjacent active sites (Figure 4A). Next, the activated *CO and *N_2_ spontaneously react both in the thermodynamic and kinetic aspects as the C-N coupling step to form a tower-like *NCON intermediate. Then, *NCONH is formed due to the first hydrogenation of *NCON. *NCOHN can be converted into *NHCONH or *NCONH_2_ through alternating or distal approaches. The generation of *NCONH_2_ has a stability of +0.14 eV, making it a better choice than *NHCONH. This step is the most energy-intensive step as a potential rate-limiting step to synthesize urea. Then, urea molecules are easy to desorb from the catalytic surface due to the exothermic subsequent reduction steps [46,47]. Table 2 shows the catalytic mechanisms of urea synthesis. In addition, efficient desorption of urea from catalyst surfaces is vital to achieve high productivity in NF. Factors, including the local reaction environment, the structure of active sites, and the interaction strength between the catalyst and urea molecules influence the mechanism of urea desorption. It is essential for effective urea desorption that there is a delicate balance in binding strength between the catalyst and urea. For example, weak binding results in premature desorption of intermediates, disrupting the completion of reaction pathways and lowering the overall yield of urea. On the other hand, strong binding causes urea molecules to remain adsorbed on the catalyst surface, blocking active sites and reducing catalytic turnover. Therefore, the design of catalysts must optimize the binding interaction to stabilize intermediates during the reaction while enabling the efficient release of urea, ensuring sustained catalytic activity, and productivity.

In addition, it is easier to couple NO_3_^−^/NO_2_^−^/NO with CO_2_ to generate urea due to a lower bond dissociation energy of N=O. As shown in Figure 4B, *CO generated by the reduction of CO_2_ reacts with *NH_2_ intermediates created from NO_3_^−^/NO_2_^−^ to form urea via the *CO-*NH_2_ coupling mechanism [48,49,50]. For example, Yu et al. (2022) reported oxide-derived core–shell Cu@Zn nanowires to synthesize urea by the *CO-*NH_2_ coupling mechanism. The obtained Faradaic efficiency was ~9.3% and the urea yield rate was ~7.3 μmol·cm^−1^·h^−1^ at −1.02 V vs. RHE. Also, *CO can react with *NO intermediates as a potential mechanism, where the presence of *OCNO and *NO intermediates are verified by in situ Sum Frequency Generation (in situ SFG) spectroscopy (Figure 4C) [51]. Moreover, *CO_2_ can react with *NO_2_ intermediates as a potential mechanism, where the presence of CO_2_NH_2_ intermediates is verified by Operando Synchrotron Radiation Fourier Transform Infrared (SR-FTIR) spectroscopy (Figure 4D), an application used in identifying functional groups and confirming the production of specific nitrogen compounds [52]. Moreover, in situ Raman spectroscopy is also capable of detecting transient intermediates and confirming the binding of nitrogen species on catalyst surfaces, which can provide insights into reaction intermediates.

## 3. Carbon-Based Catalysts

Recently, these carbon-based materials have shown great promise in improving NF performance, an area that has been extensively studied. Multiple methods have been explored to improve NF efficiency, including topology and edge-site defect engineering, metal-free heteroatom doping, and metal atomic doping (Table 3).

### 3.1. Intrinsic Defects

**Figure 5:**
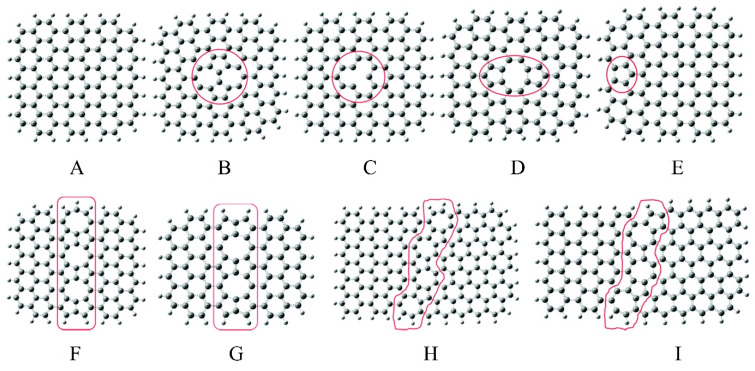
Schematic illustration of intrinsic graphene: (**A**) Perfect graphene cluster, (**B**) Stone–Wales defect (SW), (**C**) single vacancy (SV), (**D**) double vacancies (DV), (**E**) edge-defect with pentagon ring at zigzag edge (PZ), octagon, and fused pentagon carbon rings line defect with (**F**) odd number of octagon rings (GLD-558-01) and (**G**) even number of octagon rings (GLD-558-02), and pentagon–heptagon pairs line defects with (**H**) odd number of heptagon rings (GLD-57-01) and (**I**) even number of heptagon rings (GLD-57-02). The large gray ball: carbon atom; and the small white ball: hydrogen atom, respectively. Reprinted with permission from [67], copyright 2015, Royal Society of Chemistry.

The topology and edge defects are intrinsic properties of carbon materials, which contribute to their diverse functions and electronic structures. Edge defects have vacancies at the dangling groups and edges. Topological defects contain the deformations and inherent topologic vacancies at both the carbon matrix and edges [66]. Various defects (Figure 5) have been reported, including single/multiple vacancies, lattice reconstructions, non-hexagonal topologies, and dangling group [67], which significantly impact the charge density of carbon atoms close to the defects by comparing with that of carbon atoms in the basal plane. Thus, it is a potential approach to facilitate the conversion efficiency in NF. For example, Zhang et al. reported a reduced graphene oxide with tunable defects (DrGO) for NF under mild conditions in a wide pH range [53]. The NF performance of defective sites (single vacancy (SV) and double vacancy (DV) on the carbon basal plane) are examined, and an enhanced ammonia selectivity caused by the strong binding of nitrogen instead of hydrogen is performed. Density theoretical calculations show that the thermodynamic overpotential at DV sites of DrGO is similar to the most efficient transition metal-based catalysts reported so far.

### 3.2. Metal-Free Heteroatom Doping

**Figure 6:**
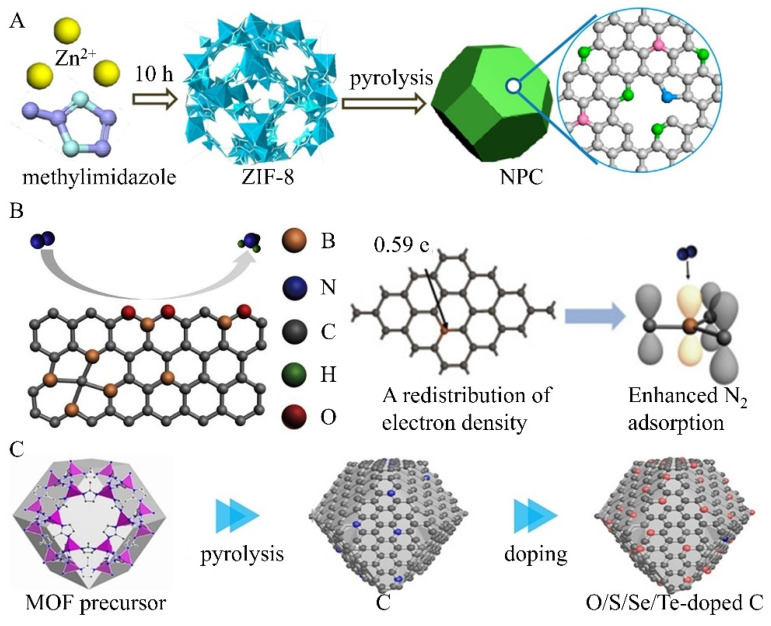
Metal-free heteroatom doping carbon catalysts: (**A**) N-doped porous carbon. Reprinted with permission from [55], copyright 2018, American Chemical Society. (**B**) B-doped graphene. Reprinted with permission from [54], copyright 2018, Cell Press. (**C**) O, S, Se, Te-doped carbon catalysts. Reprinted with permission from [76], copyright 2020, Wiley-VCH.

Another typical strategy to enhance NF performance is metal-free heteroatom doping that replaces carbon lattice atoms with heteroatoms such as chlorine (Cl), fluorine (F), sulfur (S), oxygen (O), boron (B) [54], and nitrogen (N) [40,55]. This strategy has attracted widespread attention from the materials science community [68]. It changes the spin distribution and charge density of carbon atoms, thereby affecting the adsorption behavior of products, intermediates, and reactants at particular sites [69]. In addition, it facilitates the electron transfer approach [56]. For example, nitrogen (N) has a higher electronegativity (3.04) than carbon (C, which has an electronegativity of 2.55), allowing it to steal electrons from neighboring carbons [70]. Finally, N-doping causes charge polarization and redistribution, thereby improving catalytic activity and substrate adsorption in NF under mild conditions [71]. Liu et al. pyrolyzed zeolite imidazole framework-8 (ZIF-8) to develop various nitrogen-doped porous carbon catalysts (NPC) under ambient conditions (Figure 6A) [55]. These NPCs exhibit adjustable nitrogen speciation and controllable nitrogen content (ranging from 2.1% to 13.6%) at various experimental temperatures. When applied for NRR, the NPC-750 sample with 13.6% nitrogen content achieved a maximum Faradaic efficiency (FE) of 1.42% and an ammonia yield of 1.40 mmol·h^−1^·g^−1^ at −0.9 V. Both experimental results and density functional theory (DFT) calculations highlight the pyrrolic and pyridinic nitrogen as the main active sites for nitrogen adsorption and subsequent N≡N cleavage. The preferred approach for ammonia formation involves a series of reactions: *N≡N → *NH=NH → *NH_2_–NH_2_ → 2NH_3_ [72]. Co-doping with two kinds of heteroatoms (P/N, S/N, and B/N), synergistically induces new neutral centers [73,74]. Organic precursors were polymerized to prepare metal-free co-doping carbon-based catalysts for NF [75]. Remarkable results were achieved by carefully adjusting bonding states and doping levels. N-B pairs that replace the basal plane of the graphite sheet serve as active triggers, which are verified by both experimental studies and DFT simulations. Furthermore, the edge-carbon atoms adjacent to the N-B pair serve as the catalytic site for NF. At the same time, B/N co-doping carbon-based catalysts effectively inhibit HER, as evidenced by the optimal adsorption-free-energy of *H species (0.65 eV). This carbon catalyst has an excellent FE of 13.79% and an ammonia production rate of 7.75 μg·mg_cat_^−1^·h^−1^. Yu et al. reported a B-doped graphene with a doping level of 6.2%, which performed a ${FE}_{{NH}_{3}}$ of 10.8% (at −0.5 V (RHE)) and an ammonia yield rate of 9.8 μg·mg_cat_^−1^·h^−1^ (Figure 6B) [54]. The doped boron into the graphene backbone results in a redistribution of electron density, and the electron-deficient B active sites enhance the binding affinity for nitrogen molecules. Theoretical and computational analysis demonstrate the catalytic activity of various B-doped carbon structures and identify the BC_3_ structure as having the lowest energy barrier for ammonia generation from nitrogen. Yang et al. systematically examined the performance of the oxygen/chalcogen group element (Te, Se, S, O) in the NF progress by experimental and theoretically computational analysis (Figure 6C) [76]. These doped heteroatoms accumulate the adsorption of nitrogen on the carbon atoms close to the heteroatoms. Also, Se- and Te-doped carbon catalysts show optimal NF activity, which is superior to most metal-based catalysts.

### 3.3. Metal Doping

#### 3.3.1. Single-Atom-Doped Carbon Catalysts

**Figure 7:**
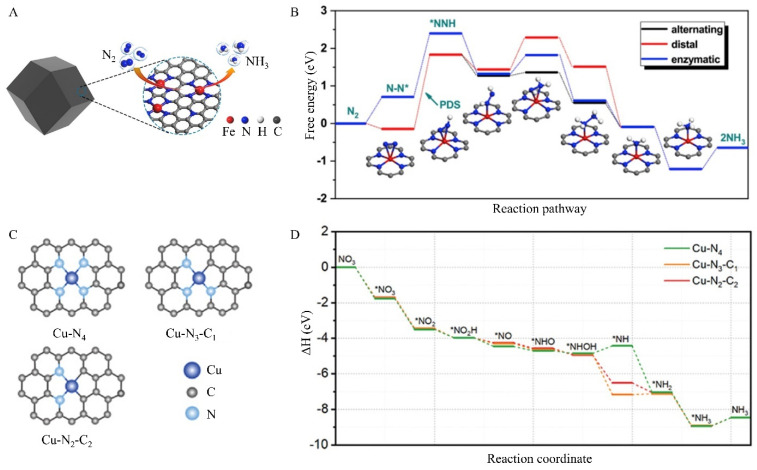
Schematic illustration of metal single-atom-doped carbon catalysts: (**A**) Fe single-atom-doped ZIF-8. (**B**) Free-energy profiles for ammonia generation on Fe single-atom-doped carbon catalysts. Reprinted with permission from [83], copyright 2019, Elsevier. (**C**) Cu single-atom-doped carbon catalyst structures. (**D**) Reaction pathway from NO_3_^−^ to NH_3_. Reprinted with permission from [84], copyright 2022, Wiley-VCH.

In addition, metal-doped carbon catalysts are a promising technology for NF [77,78]. Notably, O, S, and N can be coordinated with single-metal atoms (SA) to prepare highly reactive carbon-based single-atom catalysts (SACs). These arrangements create active centers similar to those on carbon-loaded metal-N-macrocycles as natural enzymes. Due to different dopants and metal atoms, it is easy to control the charge density of isolated metal sites, making the catalytic performance of SAC suitable for various reactions [79,80]. The earth-abundant metals (transition metals), including Cu, Ni, Co, and Fe, have been researched for E-NRR [81,82]. Fe-ZIF was carbonized to prepare single-atom Fe supported by N-doped carbon materials (FeSA/N-C) with a Fe mass loading of 4.2 wt % (Figure 7A) [83]. The EXAFS image of FeSA/N-C exhibits that the key peak at 1.5 Å assigned to the N-Fe bond, corresponding to the wavelet transform (WT) plot, in which only the N-Fe signal of FeSA/N-C is found. The maximum WT value reached is 3.7 Å^−1^. FeSA/N-C has a high ammonia formation rate of 62.9 ± 2.7 μg·mg_cat_^−1^·h^−1^ and FE of 18.6 ± 0.8%. The Fe single-atom in the Fe-N4 configuration is beneficial to the adsorption of nitrogen and subsequent activation, indicating higher E-NRR performance and catalytic selectivity, which is validated by DFT simulations. Rose’s group (2022) reported a single-atom Cu supported by N-doped carbon materials (CuSA/N-C) to synthesize urea (Figure 7B) [84]. They revealed that the Cu-N_4_ site exhibits higher activity toward CO_2_RR and that *COOH incorporation is a key parameter determining catalytic activity for urea production. Thus, the Cu-N_4_ site performs the highest ${FE}_{urea}$ of 28% at −0.9 V (RHE).

#### 3.3.2. Dual Atom-Doped Carbon Catalysts

**Figure 8:**
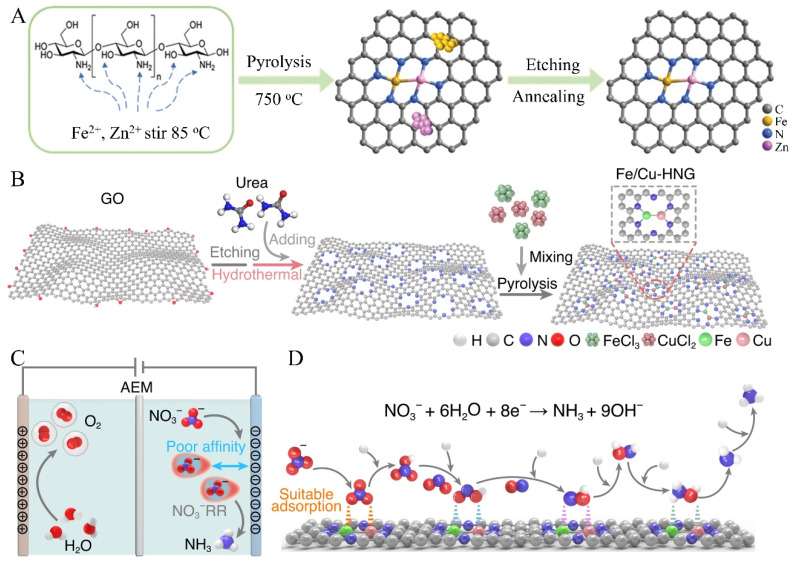
Schematic illustration of metal diatom-doped carbon catalysts: (**A**) Zn/Fe diatomic carbon catalysts. Reprinted with permission from [85], copyright 2020, Royal Society of Chemistry. (**B**–**D**) Fe/Cu diatomic graphene structure, electrochemical nitrogen reduction process, and catalytic conversion steps from NO_3_^−^ to NH_3_. Reprinted with permission from [86], copyright 2023, Nature Publishing Group.

Also, the development of metal diatomic carbon catalysts to replace SACs is vital to enhance proton coupling electron transfer. For example, Zhang et al. (2020) prepared a Zn/Fe diatomic carbon catalysts with an ${FE}_{{NH}_{3}}$ of 26.5% and a high ammonia yield rate of 30.5 μg·mg_cat_^−1^·h^−1^ (Figure 8A) [85]. Theoretical and computational analysis reveals that the Zn/Fe diatomic active sites synergistically favor nitrogen activation and reduce the reaction barrier of the intermediate formation of NNH* (the rate-limiting step). Zhang et al. (2023) reported a Fe/Cu diatomic porous graphene with a high ammonia yield rate of 1.08 mmol·mg_cat_^−1^·h^−1^ (at 0.5 V (RHE)) and a maximum ${FE}_{{NH}_{3}}$ of 92.51% (at −0.3 V (RHE)) (Figure 8B–D) [86]. The strong interactions between Fe/Cu diatomic sites and NO_3_^−^ facilitate the adsorption and discharge of NO_3_^−^ anions calculated by theoretical computation. The Fe/Cu diatomic active sites weaken the O-N bonds, resulting in the lower of the overall reaction barriers.

## 4. Electrocatalytic NF

Electrocatalytic NF (E-NF) involves the diffusion of N_2_ molecules to the working electrode (WE) surface, where they undergo electron reduction with simultaneous acquisition of protons, ultimately generating NH_3_ [87,88,89]. Compared with the traditional H-B method, E-NF has several merits, including the low energy cost, high chemical space, and sustainable proton source. (1) The traditional H-B approach relies on thermal energy, while E-NF uses electrical energy. As a result, it operates under milder conditions, making it possible to use catalysts that might not require high temperatures and pressures. (2) Liquid-phase reaction conditions in E-NRR provide a versatile chemical space for optimizing catalytic performance. By adjusting parameters such as potential range, electrolyte type, and pH, the process can be tuned. (3) The E-NF approach does not rely on fossil fuels but uses water as a proton source and reducing agent. This shift to renewable energy (wind or solar energy) enables the production of NH_3_ in a decentralized strategy. Thus, E-NF holds great promise, providing both environmental benefits and opportunities for innovative catalyst design.

E-NF unfolds within the complicated range of solid–liquid–gas three-phase interfaces. The first step involves the diffusion of N_2_ molecules onto the WE surface. These molecules are then further reduced, facilitated by electrons, while gaining protons, ultimately producing NH_3_. Nevertheless, some difficult challenges remain. For example, under ambient conditions, N_2_ solubility in water is as low as 0.66 mmol·L^−1^, which greatly restricts the production of NH_3_ [90]. In addition, the chemical inertness of N_2_ creates obstacles during adsorption and activation approaches [91]. (1) The first bond in N≡N requires a staggering 410 kJ·mol^−1^ to dissociate. (2) N_2_ has a proton affinity of ~494 kJ·mol^−1^, which lacks a constant dipole moment. Its reactivity is inhibited by the negative electron affinity (−1.90 eV) and high ionization potential (15.84 eV). (3) The electron transfer during E-NF is hindered by the small energy gap (10.82 eV) between the lowest unoccupied molecular orbital (LUMO) and the highest occupied molecular orbital (HOMO) [91]. After N_2_ adsorption/activation, subsequent reduction reactions involve complex steps, including bond breaking, hydrogenation, and electron transfer [42]. Two proposed mechanisms have emerged. (1) Association pathway: Catalysts adsorb N_2_ molecules on their surface. Next, the N≡N is destroyed by continuous hydrogenation, resulting in N-N bond cleavage and a release of NH_3_ [92]. (2) Dissociation pathway: Bond cleavage occurs with N_2_ adsorption, and then the adsorbed N atoms are independently hydrogenated to generate NH_3_ [93]. Taken together, resolving these complexities is key to efficient electrocatalytic NF and sustainable NH_3_ production.

In theory, nitrogen should convert to ammonia when a relatively negative bias voltage is applied to the electrode compared to the equilibrium barrier for E-NF (0.092 V vs. RHE) [94]. Nevertheless, this equilibrium barrier represents the average of six protons and six electrons transferred [92]. The first electron affinity of N_2_ is around −2.78 V, which emphasizes the thermodynamic difficulty of N_2_ hydrogenation [95]. Thus, the activation of nitrogen is challenging under mild conditions [96]. In contrast, only two electrons are required in parasitic HER. Only two electrons for each H_2_ are produced in one half-reaction. Protons and electrons combine easily via HER, resulting in quite low selectivity for NH_3_ (low ${FE}_{{NH}_{3}}$). To overcome these challenges, it is crucial to design carbon-based electrocatalysts in a reasonable way, which can enhance N_2_ adsorption and activation to improve E-NF activity [97].

**Figure 9:**
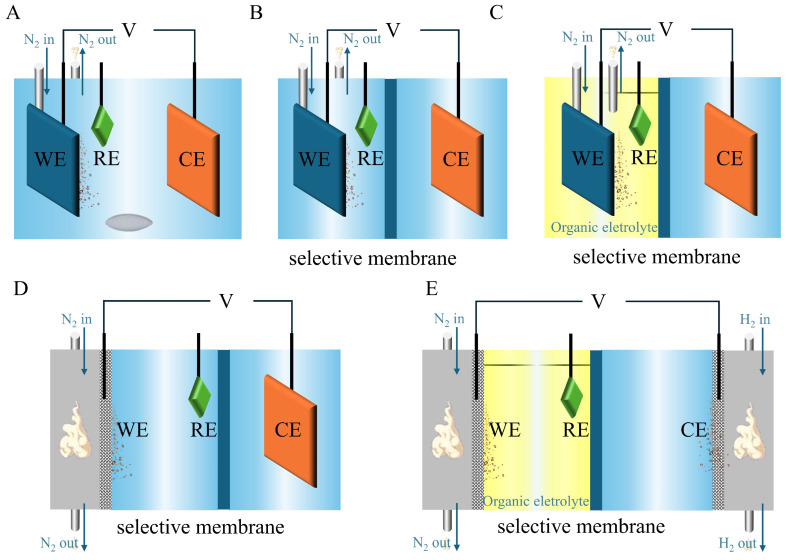
Illustration of reaction cells for E-NF: (**A**) Single-cell reactor. (**B**) H-cell reactor with a selective membrane. (**C**) H-cell reactor with a selective membrane. Organic electrolyte on the left side of the H-cell reactor and aqueous electrolyte on the right side of the H-cell reactor. (**D**) Three compartments cell reactor with a GDE. (**E**) Four compartments cell reactor with two GDE. Organic electrolyte on the left side of the H-cell reactor and aqueous electrolyte on the right side of the H-cell reactor. Reprinted with permission from [98], copyright 2024, Wiley-VCH.

Additionally, the reaction cell is a key factor affecting the catalytic performance of NF. The commonly used reaction cell for E-NF includes a single-cell reactor and, an H-cell reactor with a selective membrane, which is similar to electrocatalytic water splitting (Figure 9A–C) [98]. As shown in Figure 9A, electrodes are dipped in the electrolytes, and nitrogen is blown around WE. The single-cell reactor can also be placed in an autoclave to achieve high nitrogen saturation. A continuous stirring helps achieve a uniform distribution of the reactants [99]. The H-cell reactor (Figure 9B) has two compartments separated by a selective membrane. The cathode and anode are placed in different compartments, respectively. H_2_O and organic electrolytes can even exist in the same cell in some cases (Figure 9C) [100]. In the flow cell equipped with a gas diffusion electrode (GDE), the gas permeates via the electrode to contact the bulk electrolyte at the electrode surface, thereby establishing a three-phase boundary essential for efficient E-NF reactions (Figure 9D,E) [26]. To maintain uniform reactivity at WE, the electrolyte is typically recirculated within the flow cell, ensuring consistent ion transport and reactivity across the electrode surface.

Furthermore, it is worth mentioning that in carbon-based electrochemical nitrogen fixation methods, the reactor design and electrode configuration are crucial factors influencing the reaction efficiency. Typically, common reactors include H-type cells and flow reactors, and a carbon-based electrode array, made from materials such as graphite, carbon paper, or carbon cloth, is used due to its high surface area and excellent electrical conductivity. The electrodes are usually arranged in parallel or stacked configurations to maximize the interaction between the nitrogen gas and the electrode surface. Commonly used electrolytes are KOH, Na_2_SO_4_, and phosphate buffer. Moreover, the operating conditions, such as pH, also play a significant role in the efficiency of the reaction. A slightly acidic to neutral pH (around 6–7) is often preferred, as it helps to minimize side reactions and maintain the stability of the nitrogen reduction process. The supporting electrolyte, commonly potassium or sodium bicarbonate, is employed to enhance ionic conductivity. Moreover, the surface area of the electrodes, which can be increased through modifications like roughening, directly impacts the rate of nitrogen fixation, with a larger surface.

## 5. Photocatalytic NF

The first photocatalytic nitrogen fixation was reported in 1977, which was induced by Fe-doped TiO_2_ under UV irradiation. Holes and electrons are generated in the photocatalytic NF (P-NF) process [101]. Protons are utilized as a source of hydrogen gas, and electrons induced in the photocatalytic process are used to activate nitrogen molecules and convert them to ammonia [102]. As a comparison, water is split into hydrogen and oxygen (H_2_O → H_2_ + O_2_) [103], ΔG_298_ = 237 kJ·mol^−1^, and carbon dioxide is reduced to methane (CO_2_ + 2H_2_O → CH_4_ + 2O_2_) [104], ΔG_298_ = 237 kJ·mol^−1^ in P-NF. These reactions are difficult due to ΔG^o^ > 0, requiring high energy input by absorbing photons with energies corresponding to the shortwave visible or UV regions. NF on heterogeneous surfaces can proceed via dissociation and association mechanisms, which are the same as in E-NF. g-C_3_N_4_ has a band gap potential of ~2.7 eV to absorb visible light, which can work under solar irradiation. To improve P-NF performance, several strategies have been explored, including defect and metal doping.

In the defect strategy, an “all-in-one” strategy has been investigated to enhance the P-NF performance, particularly for g-C_3_N_4_, by introducing pores, corners/edges, noncrystallinity, strain, dopants, and vacancies [105]. This strategy could improve the response of their visible light to long-wavelength/near-infrared light range, enhance the photogenerated electron migration, decrease the photocarriers recombination, control the bandgap width to the required reduction potential of active species, tune the VB, CB position, and adjust the electron density to improve the adsorption of nitrogen on the surface of catalysts and reduction of nitrogen. Ma et al. reported an N-deficient g-C_3_N_4_ by microwaving with a P-NF rate of ~3 mg·g_cat_^−1^·h^−1^·L^−1^ [106]. Zhang et al. also prepared a C-deficient g-C_3_N_4_ by a two-step method to calcinate a bulk g-C_3_N_4_ at 500 °C for 2 h, and then calcinated the as-obtained samples at 530 °C for 2 h. XPS spectra were used to characterize the N/C atomic ratio, and XRD was employed to show the peak shift. EPR showed rich C vacancies in the final products. Its P-NF performance was ~84 mg·g_cat_^−1^·h^−1^ without any cocatalyst and sacrificial agent. Also, functional groups (e.g., cyano groups (-C≡N) [107] and amino groups (-NH_2_) [108]) can improve the P-NF performance. Wang et al. intercalated K^+^ and modified cyano groups (-C≡N) on the g-C_3_N_4_ photocatalysts [107]. The -NH_2_ in the triazine ring can react with K^+^ to generate -C≡N, contributing to an excellent ammonia formation rate of 3.42 mmol^−1^·g^−1^·h^−1^. Both experimental results and theoretical calculations proved that the -C≡N can be regenerated via a pathway analogous to the Mars van Krevelen process with the aid of the intercalated K^+^. The regenerated -C≡N not only enhanced the P-NF performance and extended the reaction cycle but also stabilized the photocatalysts. Cao et al. reported ultrathin g-C_3_N_4_ nanosheets with rich -NH_2_ groups by collecting the gaseous thermal polymerization products of urea [108]. The modified g-C_3_N_4_ nanosheets have an ammonia production rate of 60.5 µmol^−1^·h^−1^, which was almost double that of the pristine g-C_3_N_4_. Zhang et al. (2021) [109] synthesized a p-n heterojunction by Cu_2_O and g-C_3_N_4_ nanosheets, which sped up the separation of photogenerated carriers and the adsorption of visible light, leading to improved photocatalytic performance for ammonia generation. However, the loaded amount of Cu_2_O needs to be optimized because overloading Cu_2_O will cover the active sites to decrease the contact between g-C_3_N_4_ and N_2_, reducing the photocatalytic performance.

Heteroatom doping into the g-C_3_N_4_ matrix can also improve the P-NF performance. For example, Huang et al. reported an O-doped g-C_3_N_4_ matrix with an ultrahigh P-NF performance of 118.8 mg·g_cat_^−1^·h^−1^·L^−1^ under visible light irradiation [110]. Also, the O-doped g-C_3_N_4_ catalyst had excellent stability. Li et al. used the plasma method with a feeding gas of H_2_S to prepare S-doped and N defective g-C_3_N_4_ nanosheets and obtained an ammonia production rate of 6.2 mg g^−1^·h^−1^·L^−1^ [111]. Cao et al. prepared an S-doped and C-defective porous g-C_3_N_4_ nanosheets (SCNNSs) by collecting the gaseous thiourea under a self-generated ammonia atmosphere [112]. The as-obtained SCNNSs catalysts had an ammonia production rate of 5.99 mM·g_cat_^−1^·h^−1^ under the simulated solar irradiation within 4 h, which was 2.8 times higher than bulk SCN. Liang et al. used ultrathin g-C_3_N_4_ and NaBH_4_ to prepare a B-doped and N-deficient ultrathin g-C_3_N_4_ photocatalyst (BNUCN) with an ammonia production rate of 435.28 µmol^−1^·g^−1^·h^−1^ under visible light [113]. The significant enhancement in P-NF can be attributed to (1) enhanced nitrogen adsorption capacity, photocarriers separation efficiency, and visible light absorption due to the N-deficient in g-C_3_N_4_ after doping B; (2) the doped B atoms can improve the adsorption of nitrogen and then enhance the activation of nitrogen on the catalyst surface; and (3) after doping B, the -C≡N was generated and can improve the P-NF performance. The length of N-N of nitrogen molecules was extended, further improving the adsorption energy of nitrogen on the catalysts, and increasing ammonia yield, which was verified by theoretical calculations.

In addition, the doped transition metal single atoms can improve the P-NF performance by activating nitrogen molecules and boosting visible light absorption. The empty bonding orbitals of the nitrogen molecule can accept electrons in the bonding orbitals of the nitrogen molecule, while the electrons occupied by the d-orbitals in the TM can be transferred to the antibonding orbitals of the nitrogen molecule. Thus, TM can weaken the bond strength of nitrogen molecules, which is favorable to nitrogen activation. Fe-EDTA-CNNS was prepared by grafting ethylenediaminetetraacetic acid (EDTA) on g-C_3_N_4_ nanosheets and further chelating Fe^3+^, which has a high P-NF performance of 50 µmol^−1^·h^−1^·L^−1^ [114]. The doped Fe was highly dispersed and stable. The Fe-EDTA-CNNS catalysts have high charge separation and transfer capacity, and an enhanced absorption for visible light, compared to the un-modified samples. Liu et al. reported that the single-atom Co-doped g-C_3_N_4_ catalysts had an ammonia production rate of 50.2 µmol^−1^·h^−1^ with a stable performance on cycling [115]. The ammonia production rate of single-atom Co-doped g-C_3_N_4_ catalysts was six times that of bulk g-C_3_N_4_ catalysts. Although bismuth-, metal–organic frameworks (MOFs)-, and copper-based nanomaterials have a good photocatalytic performance to remove pollution in aquatic environments via specific functionalization, carbon nanomaterials have good photocatalytic performance and show strong competition in NF (Table 4) [116,117,118].

**Figure 10:**
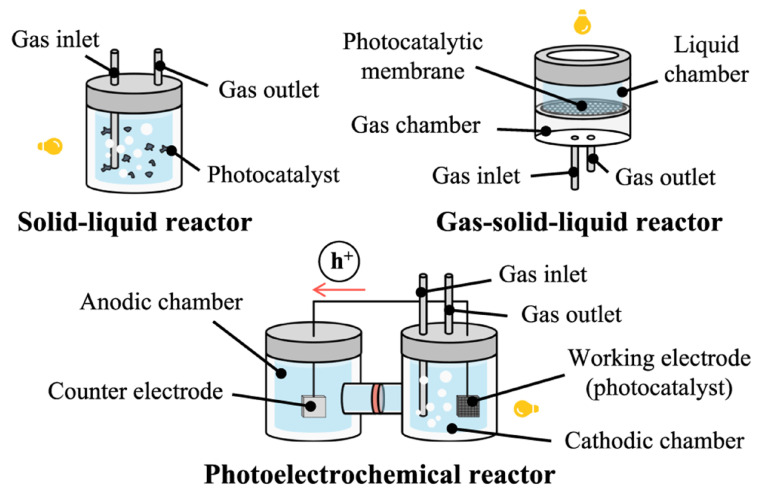
Illustration of reaction cells for P-NF. Reprinted with permission from [119], copyright 2022, Nature Publishing Group.

P-NF is typically conducted in heterogeneous suspension systems, where solid powder photocatalysts are dispersed directly into an aqueous solution. This solution may consist of pure water or water containing added hole scavengers, such as methanol or ethanol, to enhance the reaction efficiency. The design of reaction cells for P-NF shares similarities with those used in E-NF (Figure 10) [119]. In these systems, a continuous flow of nitrogen gas is introduced, facilitating contact between the nitrogen molecules, photocatalyst particles, and the aqueous medium to drive the reaction.

Also, it is worth noting that the applied solution works as the whole sacrificial agent and significantly impacts NF. As reported by Muhammad et al. (2024), NaHCO_3_ can consume some holes to inhibit urea oxidation, and some holes can oxidize water to provide protons for urea synthesis [120]. However, the amount of NaHCO_3_ used also needs to be strictly controlled. Compared with 0.4 M NaHCO_3_, 0.2 M NaHCO₃ has a reduced ability to quench photogenerated holes, thereby enhancing water and urea oxidation. This is evidenced by the higher oxygen yield and lower urea yield at 0.2 M NaHCO_3_ compared with 0.4 M NaHCO_3_. At 0.8 M NaHCO_3_, the increased consumption of holes by NaHCO_3_ resulted in a significant decrease in oxygen yield, while the urea yield was lower than that at 0.4 M NaHCO_3_. This decrease may be attributed to the reduced availability of protons for water oxidation. The highest urea yield was obtained at 0.4 M NaHCO_3_, indicating that it achieved an optimal balance between suppressing excessive urea oxidation and maintaining efficient water oxidation, thereby ensuring sufficient proton supply for urea synthesis. However, the amount used also needs to be strictly controlled.

**Table 4:** Reported photocatalysts for NF.

	Catalyst	Yield Rate	AQE	Ref.
NH_3_	0.6Cu_2_O/CN	500 μmol·h^−1^·mg_cat_^−1^	0.57%	[109]
	Cu_2_O	-	0.1%	
	Pt_1_-Pt_n_-TiN	637 μmol·h^−1^·mg_cat_^−1^	0.1%	[121]
	g-C_3_N_4_-V	84 μmol·h^−1^·mg_cat_^−1^	-	[122]
	V_N_-g-C_3_N_4_	5.5 mg·L^−1^·h^−1^·mg_cat_^−1^	-	[123]
	Co-g-C_3_N_4_	5.8 mg·L^−1^·h^−1^·mg_cat_^−1^	-	[124]
	SiW_12_/K-C_3_N_4_	353.2 μmol·h^−1^·mg_cat_^−1^	-	[125]
	c-PAN/Bi_2_OW_6_	160 μmol·h^−1^·mg_cat_^−1^	-	[126]
CO(NH_2_)_2_	SrTiO_3_-FeS-CoWO_4_	8054.2 μg·h^−1^·mg_cat_^−1^		[120]
	Ru-TiO_2_	24.95 μmol·h^−1^·g^−1^	4.7% at 380 nm	[127]
			6.3% at 420 nm	

Tips: AQE: apparent quantum efficiency. Pt_1_-Pt_n_-TiN: single atoms and clusters of platinum on TiN. g-C_3_N_4_-V: porous white powders with surface carbon vacancies. V_N_-g-C_3_N_4_: N vacancies doped C_3_N_4_. c-PAN/Bi_2_OW_6_: cyclized polyacrylonitrile (c-PAN) decorated on Bi_2_WO_6_. “-” indicates that the corresponding parameter was not provided in the given reference.

## 6. Conclusions and Outlook

This review presents a thorough summary of carbon-based catalysts for NF. For carbon materials doped with heteroatoms, neighboring heteroatoms with weak or strong electron affinities can lead to a re-distribution of charge and spin density in the carbon skeleton, resulting in non-electron neutral sites. Non-electron-neutral carbon or heteroatoms can act as Lewis acids to promote nitrogen adsorption (a precursor step to NF) and inhibit HER, thereby increasing the efficiency of ammonia conversion. Doping strategies help increase the density of active sites, thereby improving the NF performance. C atoms on the basal plane have an obvious difference in charge density compared to C atoms close to the defects of carbon catalysts with topological defects and edge sites, thus increasing their NF performance. In SACs, metal single atoms distributed atomically can be ligated with N (N-M) or other heteroatoms (e.g., S-M and B-M) in the carbon matrix. The unsaturated coordination situation results in a large amount of charge transfer from SAC to carbon materials, resulting in a high efficiency for NF.

Research for NF has made significant progress, but there are still some limitations and challenges that need to be resolved to achieve practical applications. (1) Several electrocatalysts exhibited optimal FE (>50%), but high selectivity just occurred at low overpotentials and low ammonia production rates. FE reduces with an increase in negative potential where HER dominates. SACs can improve selectivity for urea by offering isolated active sites that reduce HER competition. Also, the apparent quantum efficiency (AQE) values can be used instead of the FE to compare the photocatalytic performance when a n- or p-type semiconductor is employed. (2) The NF performance of SACs is impacted by ligand effects. Nevertheless, harsh reaction conditions (e.g., high temperatures) inhibit the introduction of functional groups to support SAC. Thus, it is necessary to develop simple preparation and optimization methods to synthesize SACs. (3) The current understanding of the mechanism depends heavily on DFT simulations, but NF in solution is very complex. FE may not always accurately reflect the performance of nitrate reduction reactions due to competing reactions such as the HER. Thus, there is a strong need to study mechanisms by spectroscopic techniques to detect intermediates to directly verify nitrogen fixation products and distinguish them from HER byproducts. The in situ characterizations include in situ Raman spectroscopy, gas chromatography–mass spectrometry (GC-MS), in situ Fourier transform infrared spectroscopy (FTIR), in situ Sum Frequency Generation (in situ SFG), and spectroscopy (UV–Vis spectroscopy). Electrochemical techniques can also be applied, including in-situ electrochemical impedance spectroscopy (EIS) and differential electrochemical mass spectrometry (DEMS). Also, the isotopic labeling can work as a suggested complementary characterization technique. With these characterizations, it is good to monitor the formation of intermediates such as *NH_2_, *CO, and *NCON, leading to optimizing the output performance of NF. (4) Urea exhibits strong adsorption on many catalysts, which can inhibit its desorption and reduce overall productivity. This is particularly problematic in systems where the catalyst binds strongly to the reaction intermediates. Modifying the surface structure of catalysts (e.g., introducing defects or functional groups) can facilitate urea desorption and minimize undesired adsorption effects. (5) The catalyst leaching and deactivation during prolonged operation hinder the durability and stability of these catalysts. Biomimetic Catalysts inspired by nitrogenase enzymes can replicate natural pathways for urea production, offering improved efficiency.

The adsorption intensity between intermediates/reactants and metal sites impacts the NF selectivity of these catalysts. Novel design methods can fine-tune the coordination environment, thereby optimizing the adsorption intensities. (1) The development of diatomic catalysts with heteroatom and various metal atoms coordination, inspired by the structures of biological nitrogenases (e.g., FeV and FeMo), is a promising strategy. (2) Metal single atoms coordinated carbon materials strategy is another potential method, which favors electron localization and modulates the adsorption intensities of intermediates and reactants. (3) The defective structure of SAC-embedded carbon-based materials can be expanded to promote E-NRR and inhibit competitive HER. To date, research on the various coordination conditions of the central metal has been limited. In the coming future, progress in these strategies will lead to a higher ammonia production rate and FE, thereby enabling the replacement of the fossil fuel-dependent and energy-intensive H-B process by the NF process. In addition, unlike metal alloys, which often face segregation challenges that impact their performance, heteroatom-doped carbon-based nanomaterials show excellent catalytic performance. This operation stability arises from the strong covalent bonds formed between the incorporated dopants and carbon atoms, effectively mitigating segregation. Also, the strategic co-doping of carbon nanomaterials with various heteroatoms (e.g., S, B, N, and F) enables the design of catalysts with a wide spectrum of active sites, enhancing the versatility of their catalytic performance. Thus, it is vital to design novel carbon-based catalysts for NF.

Furthermore, the influence of semiconductor types on the electronic surface state and reaction pathways should also be taken seriously. N-type semiconductors tend to facilitate electron transfer due to their abundance of free electrons, potentially enhancing the reduction of nitrogen to nitrogenous species. This can also shift the reaction pathway toward more selective nitrogen reduction. P-type semiconductors often exhibit strong hole-induced oxidation effects, which may influence the adsorption and activation of nitrogen molecules on the catalyst surface, thereby impacting the overall reaction pathway. The electronic structure of the semiconductors (e.g., bandgap, conduction, and valence band positions) also plays a crucial role in modulating catalytic activity and selectivity.

## Figures and Tables

**Table 1:** The detailed intermediates occurring in various approaches to ammonia generation.

Mechanism Routes	Elementary Reaction Steps
Dissociative	N_2_ + 2* → 2*N
	*N + e^−^ + H^+^ → *NH
	*NH + e^−^ + H^+^ → *NH_2_
	*NH_2_ + e^−^ + H^+^ → NH_3_ + *
Associative distal	N_2_ + * → *N_2_
	*N_2_ + e^−^ + H^+^ → *NNH
	*NNH + e^−^ + H^+^ → *NNH_2_
	*NNH_2_ + e^−^ + H^+^ → *N + NH_3_
	*N + e^−^ + H^+^ → *NH
	*NH + e^−^ + H^+^ → *NH_2_
	*NH_2_ + e^−^ + H^+^ → NH_3_ + *
Associative Alternating, and enzymatic	N_2_ + * → *N_2_
	*N_2_ + e^−^ + H^+^ → *NNH
	*NNH + e^−^ + H^+^ → *NHNH
	*NHNH + e^−^ + H^+^ → *NHNH_2_
	*NHNH_2_ + e^−^ + H^+^ → *NH_2_NH_2_
	*NH_2_NH_2_ + e^−^ + H^+^ → *NH_2_ + NH_3_
	*NH_2_ + e^−^ + H^+^ → NH_3_ + *

**Table 2:** The catalytic mechanisms for generating nitrogenous compounds.

Compounds	Mechanism	
Urea	CO_2_ + N_2_ + 6H^+^ + 6e^−^ → NH_2_CONH_2_ + H_2_O	E^0^ = 0.211 V
	CO_2_ + 2NO + 10H^+^ + 10e^−^ → NH_2_CONH_2_ + 3H_2_O	E^0^ = 0.772 V
	CO_2_ + 2NO_2_^−^ + 16H^+^ + 14e^−^ → NH_2_CONH_2_ + 5H_2_O	E^0^ = 0.833 V
	CO_2_ + 2NO_3_^−^ + 18H^+^ + 16e^−^ → NH_2_CONH_2_ + 6H_2_O	E^0^ = 0.811 V

**Table 3:** Performance of recent carbon-based catalysts.

	Catalyst	Electrolyte	Yield Rate	FE	Ref.
NH_3_	DrGO	0.1 M KOH	7.4 μg·h^−1^·mg^−1^	10.8%	[53]
		0.1 M HCl	7.8 μg·h^−1^·mg^−1^	22.0%	
	BG	0.05 M H_2_SO_4_	9.8 μg·h^−1^·cm^−2^	10.8%	[54]
	N-PC	0.05 M H_2_SO_4_	23.8 μg·h^−1^·mg_cat_^−1^	1.42%	[55]
	P-G	0.5 M LiClO_4_	32.33 μg·h^−1^·mg_cat_^−1^	20.82%	[56]
	TiO_2_@C	0.1 M Na_2_SO_4_	20.03 μg·h^−1^·mg_cat_^−1^	10.76%	[57]
	VO_2_@CN	0.1 M Na_2_SO_4_	0.31 μmol·h^−1^·mg_cat_^−1^	67.9%	[1]
		0.1 M HCl	0.52 μmol·h^−1^·mg_cat_^−1^	61.9%	
	Fe SAC/N-C	0.1 M KOH	53.13 μg·h^−1^·mg_cat_^−1^	39.6%	[58]
	a_1_-Ru/CNTs	5 mM Cs_2_CO_3_	10.49 μg·h^−1^·mg_cat_^−1^	17.48%	[59]
	Fe_SAC_-N-C	0.1 M KOH	3.47 μg·h^−1^·cm^−2^	23.7%	[60]
	Fe_SA_-N-C	0.1 M KOH	7.48 μg·h^−1^·mg_cat_^−1^	56.55%	[61]
CO(NH_2_)_2_	Fe(a)@C-Fe_3_O_4_/CNTs	0.1 M KNO_3_	1341.3 μg·h^−1^·mg_cat_^−1^	16.5%	[62]
	CuPc-Amino	0.1 M KHCO_3_	103.1 mmol·h^−1^·g^−1^	11.9%	[63]
	N-doped C	0.1 M KHCO_3_	102.2 mg·h^−1^·mg_cat_^−1^	0.55%	[64]
CH_3_NH_2_	CoPC-NH_2_/CNT	0.1 M KHCO_3_	-	13%	[65]

Tips: DrGO: defective reduced graphene oxide. BG: B-doped graphene. N-PC: N-doped porous carbon. P-G: P-doped graphene. TiO_2_@C: TiO_2_ decorated juncus effusus-derived carbon microtubes. VO_2_@CN: VO_2_ anchored on N-doped carbon. Fe SAC/N-C: Fe single-atom catalyst (SAC) anchored on N-doped carbon. a_1_-Ru/CNTs: ultrafine amorphous Ru nanoclusters supported on CNTs. Fe_SAC_-N-C: Fe single-atom immobilized on nitrogen-doped carbon nanosheets. Fe_SA_-N-C: single-atom dispersed Fe-N-C. Fe(a)@C-Fe_3_O_4_/CNTs: symbiotic graphitic carbon encapsulated amorphous iron and Fe_3_O_4_ nanoparticles on carbon nanotubes. CuPc-Amino: copper phthalocyanine strengthened by amino substitution. CoPC-NH_2_/CNT: cobalt β-tetraaminophthalocyanine/carbon nanotube. “-” indicates that the corresponding parameter was not provided in the given reference.

## Data Availability

Data sharing is not applicable. No new data were created or analyzed in this study.
